# Remote ischemic conditioning (RIC) with exercise (RICE) is safe and feasible for acute ischemic stroke (AIS) patients

**DOI:** 10.3389/fneur.2022.981498

**Published:** 2022-11-15

**Authors:** Yanna Tong, Hangil Lee, Wesley Kohls, Zhenzhen Han, Honglian Duan, Zhe Cheng, Fenghai Li, Jie Gao, Jing Liu, Xiaokun Geng, Yuchuan Ding

**Affiliations:** ^1^Institute of Neuroscience, Beijing Luhe Hospital, Capital Medical University, Beijing, China; ^2^Department of Neurology, Beijing Luhe Hospital, Capital Medical University, Beijing, China; ^3^Department of Neurosurgery, Wayne State University School of Medicine, Detroit, MI, United States

**Keywords:** thrombolysis and thrombectomy, early rehabilitation, exercise rehabilitation, neuroprotection, remote ischemic conditioning

## Abstract

**Objective:**

Rehabilitation is essential in reducing stroke disability and should be performed as early as possible. Exercise is an established and effective rehabilitation method; however, its implementation has been limited as its very early use exacerbates cerebral injury and is restricted by patients' unstable conditions and disabilities. Remote ischemic conditioning (RIC) is a passive and accessible therapy in acute phases of stroke and appears to have similar neuroprotective effects as exercise. This study assessed the safety and feasibility of the novel rehabilitation strategy—early RIC followed by exercise (RICE) in acute ischemic stroke (AIS).

**Methods:**

We conducted a single-center, double-blinded, randomized controlled trial with AIS patients within 24 h of stroke onset or symptom exacerbation. All enrolled patients were randomly assigned, at a ratio of 1:1, to either the RICE group or the sham-RICE group (sham RIC with exercise). Each group received either RIC or sham RIC within 24 h after stroke onset or symptom exacerbation, once a day, for 14 days. Both groups started the exercise routine on day 4, twice daily, for 11 total days. The safety endpoints included clinical deterioration, recurrence of stroke, hemorrhagic transformation, complications, and adverse events resulting from RICE during hospitalization. The efficacy endpoints [Modified Rankin Scale (mRS) score, National Institutes of Health Stroke Scale (NIHSS) score, Barthel Index, and walking ability] were evaluated at admission and 90 days after stroke onset.

**Results:**

Forty AIS patients were recruited and completed the study. No significant differences in baseline characteristics were found between the two groups, which included risk factors, stroke severity at admission, pre-morbid disability, and other special treatments. No significant differences were found in the safety endpoints between two groups. Excellent recovery (mRS 0–2) at 3 months was obtained in 55% of the patients with RICE as compared 40% in sham group, but it did not reach a significant level.

**Conclusions:**

RICE was safe and feasible for AIS patients, and seems to be a promising early stroke rehabilitation. The results of this study suggest a need for a future randomized and controlled multicenter trial with a larger sample size to determine the efficacy of RICE.

## Introduction

Ischemic stroke remains a leading cause of death and disability worldwide (1). Advances in medical technology (including thrombolysis, endovascular intervention, and life support) have significantly reduced the mortality of stroke patients (2, 3), while persistently increasing the disability rate (4). Thus, the need for effective stroke rehabilitation is likely to remain an essential part of the continuum of stroke care for the foreseeable future (5). However, the optimal window for neuroplasticity to positively affect motor recovery only spans the early stage following a stroke (6); therefore, interventions that promote early neuroplasticity have the greatest potential to improve motor recovery (7). The latest stroke rehabilitation guidelines advocate for early rehabilitation (5), but there is no conclusion on the appropriate rehabilitation training strategy (8), which has become a barrier to practical clinical rehabilitation.

Physical exercise has long been considered a fundamental strategy for stroke rehabilitation (5, 9). Multiple studies have confirmed that exercise can reduce brain injury (10), facilitate synaptogenesis and neuroplasticity, improve motor function as well as cognition, and reduce the degree of disability (11, 12). In light of the broad benefits of exercise in stroke patients, physical exercise is at the forefront of early rehabilitation (10). However, certain challenges exist in implementing exercise therapy in acute stroke. A Very Early Rehabilitation Trial (AVERT) showed that very early mobilization (<24 h) after stroke exacerbated brain injury and reduced rates of favorable prognoses measured at 3 months (13). We found similar results in our clinical study on very early mobilization (14). Furthermore, intravenous thrombolysis, mechanical thrombectomy, and other life-saving measures take precedence in the hyperacute post-stroke setting and are often not amenable to adjuvant physical therapy (4). Additionally, patients at the early stages of stroke recovery had unstable body conditions and poor fitness, making it challenging to adapt to exercise training, thereby limiting the promotion of early exercise rehabilitation poststroke (9). Currently, there is an unoccupied period of rehabilitation that spans the early stages of stroke recovery, which leaves the theoretical benefits of early rehabilitation largely unfulfilled. Therefore, it is urgent and necessary to explore accessible rehabilitation models that can be implemented safely at the early stage of stroke recovery.

RIC is a noninvasive, low-cost, and well-tolerated novel neuroprotective therapy (15). It works through controlled and transient periods of subcritical ischemia to non-vital arteries (16–18), activating endogenous tissue repair mechanisms to exert neuroprotective effects, cardiovascular protection, and promote neurological recovery (4, 19, 20). Recently, Chen and his colleagues published the results of the Remote Ischemic Conditioning for Acute Moderate Ischemic Stroke (RICAMIS) trial, the largest randomized clinical trial to date of RIC in patients with AIS (21). This study indicated that 2 weeks of RIC in patients with acute moderate ischemic stroke within 48 h from symptom onset was associated with an increase in the odds of a favorable outcome (modified Rankin Scale [mRS] 0–1) at 90 days (21, 22). The result of this trial demonstrated that RIC training at the acute stage of ischemic stroke was feasible and improved long-term functional prognosis. Unlike exercise, it is less dependent on the patient's mobility, disability, and level of motivation, and thus can be applied to the patient passively (23). Current evidence suggests that RIC has the potential to confer similar neuroprotective effects as exercise with a broader temporal window, including the hours immediately following stroke (24). Clinical studies have confirmed that RIC is safe and feasible within 24 h after stroke, including in patients with intravenous thrombolysis or mechanical thrombectomy, which indicates that, unlike exercise, it is amenable to and does not interfere with the standard treatment protocol of stroke (15). Therefore, RIC appears to be a suitable strategy for the hyperacute and very early acute phases of stroke recovery, when patients may be unable to tolerate intensive exercise or rehabilitation protocols (25).

The complementary benefits and pitfalls of exercise and RIC suggest that they could be combined into an optimized combination therapy for AIS patients. Thus, we developed a novel early stroke rehabilitation model, the RICE, in which RIC is initiated soon after ischemic stroke and subsequently followed by physical exercise, maximizing the therapeutic potentials of both therapies (4, 26). As the first step, the current study aimed to investigate the safety and feasibility of RICE for AIS patients. The results of this study will help plan a future phase II study to establish RICE as a rehabilitative strategy for stroke patients.

## Subjects and methods

### Study design

This study was a single-center, double-blinded, randomized controlled trial registered on Clinicaltrials.gov (ChiCTR2000041042). The study protocol and informed consent were approved by the Ethics Committee of Beijing Luhe Hospital, Capital Medical University. All patients were consecutive patients hospitalized in Beijing Luhe Hospital from December 1, 2020 to December 31, 2021. All patients enrolled were randomly assigned to either the RICE group (RIC followed by exercise) or the sham-RICE group (sham RIC followed by exercise) at a ratio of 1:1, with 20 patients in each group. The primary outcomes were assessed through safety endpoints, including clinical deterioration, recurrence of stroke, hemorrhagic transformation, complications, and adverse events resulting from RICE during hospitalization. The secondary outcomes were evaluated through efficacy end-points, which included the mRS score, NIHSS score, Barthel Index, and walking ability. Full details of the study rationale, design, and statistical analysis have been published elsewhere (26).

### Patient population

Eligible patients were aged 18–80 years old, had confirmed acute ischemic stroke (mRS ≤ 2 and NIHSS score: 6–16), and were recruited from hospital wards and randomized grouping within 24 h of the ischemic event or symptom exacerbation. The criteria for exclusion were as follows: (1) contraindications for ischemic conditioning (e.g., severe soft tissue injury, fracture, and peripheral vascular disease in both upper limbs), (2) coagulation dysfunction or active bleeding; (3) unstable vital signs (e.g., systolic blood pressure <120 or >220 mmHg, heart rate <40 beats/min or >100 beats/min, percutaneous oxygen saturation ≤ 92%, body temperature ≥38.5°C); (4) severe hepatic, pulmonary, and/or renal dysfunction; (5) lower limb fracture(s) or other factors that would prevent exercise training completion; (6) combined acute coronary syndrome or severe arrhythmia; (7) pregnant or lactating patients; (8) life expectancy ≤ 1 year; (9) history of poor compliance; and/or (10) participation in another clinical trial currently or within 30 days before study inclusion. Informed consent was obtained from all participants or a legally authorized representative.

### Randomization

The enrolled patients were randomly assigned (1:1), to either the intervention group (RICE group) or the control group (sham-RICE group). A predefined table was generated by a computer program to make randomized sequence column orders, and the sequence was hidden in an enclosed opaque envelope. The baseline patient data was collected by a specialized member of the research team, and then subjects were randomly assigned to the RICE group or sham-RICE group.

### Interventions

Based on the research protocol (26), an electric autocontrolled device (Patent No. CN200820123637.X, China) was used in this study for both RIC and sham RIC. Both RIC and sham RIC were performed by using the same electric autocontrolled device, preventing discernment of the patient's group assignment by the device's appearance. The electric autocontrolled device was programed to run two different protocols named “Program 1” and “Program 2,” which provided RIC and sham RIC, respectively. The inflation pressure was set to 200 mmHg for the RIC group (Program1) and 60 mmHg for the sham-RIC group (Program 2). Only the lead investigator, who did not partake in training and evaluation of patients, was aware of the programming. RIC was completed by wrapping an electronic tourniquet around each arm within 24 h of stroke onset or symptom exacerbation. The RICE group patients received five cycles of cuff inflation to 200 mmHg for 5 min, followed by cuff deflation for an additional 5 min. The procedure was repeated once daily for a total of 14 days. The sham-RICE group underwent the same procedure as the RICE group, but the inflation pressure was set to 60 mmHg. The process for the sham-RICE group was also repeated daily for 14 days.

Starting 4 days after symptom onset, each group was given out-of-bed exercise training two times daily for 30 min, for a total of 11 days. The out-of-bed mobilization included sitting, standing, and walking, and was completed with or without assistance as described by the “A Very Early Rehabilitation Trial” (AVERT) Protocol. Briefly, the patient was assisted and encouraged in functional tasks, including activities such as sitting over the edge of the bed, standing up, sitting out of bed, and walking. No specialized equipment was used for mobilization, but common assistive equipment (e.g., standing beds, standing hoists, standing frames, tilt tables, wheelchairs, lap trays, gait aids, arm supports, safety belts, etc.) were allowed when needed. The mobilization protocols were performed by professional therapists or nurses, and were carried out based on each individual patient's needs, tolerances, and abilities and adjusted with recovery. For example, poorer-functioning patients (patients with higher NIHSS scores) performed sitting or standing tasks with the help of professional therapists or nurses while using wheelchairs or standing beds. Higher-functioning patients (patients with lower NIHSS scores) performed standing or walking tasks with little help or without help. An assigned staff member monitored the quantity and quality of exercise to ensure proper compliance for this study.

**Figure 1:**
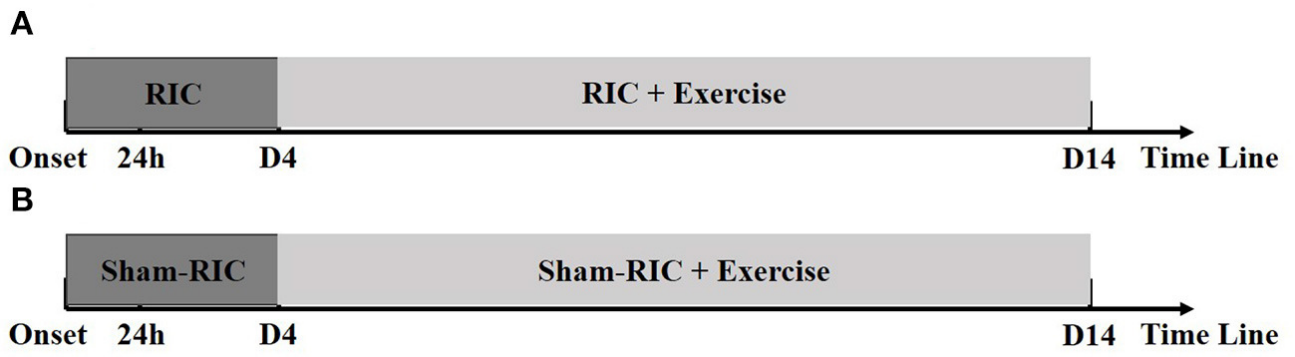
Timelines for experimental procedures. **(A)** RICE group (RIC with exercise); **(B)** Control group (Sham-RIC with exercise).

Physicians monitored patients for deteriorating conditions during exercise, and were instructed to postpone mobilization when appropriate. All patients were treated based off the standard stroke treatment guidelines including thrombolysis, anti-platelet aggregation, and lipid reduction (Figure 1).

### Outcomes

Primary outcome: Safety was the primary outcome measured in this study, which included all serious RIC/sham-RIC-related and exercise-related complications and adverse events. The other safety endpoints included hemorrhagic transformation, recurrence of stroke, clinical deterioration, myocardial infarction, angina, chest infection, pulmonary embolism, deep venous thrombosis, urinary tract infection, pressure sore, and fall during hospitalization. Specially trained members of the research group, who were blinded to the randomization and intervention programs and did not participate in other parts of the study, performed security evaluation daily during the patients' hospitalization and determined all complications and adverse events. The source data was reviewed by the research team when appropriate.

Secondary outcome: Efficacy was the secondary outcome measured in this study, which was defined by the mRS (mRS 0–2 points was classified as a favorable prognosis, while mRS 3–6 points was defined as a poor prognosis), NIHSS score, Barthel Index, and the proportion of patients achieving independent walking after 90 days (Holden functional classification of walking). These measures were evaluated as indicated by 3 specially trained members of the research group, who were blinded to the randomization and intervention programs.

Based on the protocol (26), we assigned a special evaluation team composed of 3 trained members, who were blinded to the randomization and intervention programs and not participating in other parts of the study, to evaluate the clinical endpoints.

### Sample size estimation

This study was a phase I safety and feasibility trial. There was no data available to reference as there had been no clinical studies of RIC and exercise therapy in patients with confirmed AIS (mRS ≤ 2, NIHSS score: 6–16). Although, Hertzog suggested that 10–20 patients in each group are adequate to assess the feasibility of a pilot study (27). Dobkin showed that 15 patients in each group is typically enough to determine if it is warranted to conduct a larger multicenter trial (28). We set the recruitment goal to 20 patients in each group to conform with both recommendations (26). The results of this study will help determine the safety and feasibility of RICE, as well as help estimate the sample size and calculate the power for a future phase II trial.

### Statistical analysis

The statistical software Statistical Analysis System (SAS, version 9.1.3: SAS Institute, Cary, NC) was used to perform all statistical analysis. Outcome event analyses were based on the intention to treat principle (ITT), which included all randomly enrolled subjects. *P* < 0.05 was considered significant. Demographic and clinical characteristics were analyzed through descriptive statistics. Continuous data was presented as a mean (standard deviation) and categorical data was presented as a number and percentage. Continuous variables in normal distributions were compared by the independent samples *t*-test or analysis of variance (ANOVA), while non-normal distribution continuous variables were compared by the rank sum test. Categorical variables were compared using chi-square testing.

## Results

From December 1, 2020 to December 31, 2021, 40 consecutive patients hospitalized in Beijing Luhe Hospital were randomly assigned (1:1) to the two groups. All of the patients finished the training and follow-up assessment.

### Baseline characteristics

Baseline characteristics including age, gender, affected limb, time to first RIC/RIC-sham, time to first exercise, risk factors, and pre-morbid disability were similar between the two study groups. There was no significant difference of stroke severity between the two groups based on NIHSS score and Barthel index. The majority of patients had a first-time ischemic stroke (85.0% in both groups) and all the enrolled patients had moderate strokes with NIHSS scores between 6 and 16. A subset of patients received special clinical treatments, such as intravenous thrombolysis (20% in RICE, 25% in sham-RICE), mechanical thrombectomy (20% in RICE, 20% in sham-RICE), or bridging (intravenous thrombolysis and mechanical thrombectomy) (45% in RICE, 35% in sham-RICE) in the hyperacute phase of stroke before implementation of our rehabilitation strategy. There were no significant differences in the demographic characteristics between the two groups in this specific patient population. Details of the baseline characteristics are displayed in Table 1.

**Table 1:** Baseline characteristics of the patients.

	**RICE (RIC** ** with exercise)** ** (*n =* 20)**	**Sham-RICE (Sham** ** RIC with** ** exercise)** ** (*n =* 20)**	***P*-value**
**Basic elements**			
Age (years)	62.9 ± 9.1	60.6 ± 14.2	0.538
Sex (male)	13 (65%)	12 (60%)	0.744
Affected limb (left)	12 (60%)	11 (55%)	0.749
Time to first RIC/ RIC-Sham (h)	17.5 ± 6.0	17.9 ± 6.9	0.828
Time to first exercise	86.7 ± 5.9	87.1 ± 5.0	0.818
**Risk factors**			
Hypertension Diabetes mellitus	17 (85%) 10 (50%)	15 (75%) 7 (35%)	0.693 0.337
Hypercholesterolemia	14 (70%)	13 (65%)	0.736
Ischemic heart disease	4 (20%)	4 (20%)	1.000
Atrial fibrillation	3 (15%)	5 (25%)	0.693
Previous stroke or TIA	3 (15%)	3 (15%)	1.000
Smoking	11 (55%)	7 (35%)	0.204
Alcohol drinking	11 (55%)	9 (45%)	0.527
**Stroke severity**			
NIHSS score	9.4 ± 3.1	10.9 ± 3.6	0.149
Barthel index	16.8 ± 12.3	18.0 ± 12.3	0.749
**Pre-morbid disability**			0.971
mRS 0	19 (95%)	19 (95%)	
mRS 1	0	1 (5%)	
mRS 2	1 (5%)	0	
**Other special treatments**			
rtPA treatment	4 (20%)	5 (25%)	1.000
Endovascular thrombectomy	4 (20%)	4 (20%)	1.000
Bridging	9 (45%)	7 (35%)	0.519

### Safety

No serious RICE or sham-RICE related adverse events occurred. Although more patients in the RICE group experienced skin petechiae in the superior part of the upper arm (80% in RICE, 10% in sham-RICE), all participants in both groups tolerated the RICE/sham-RICE procedures without discomfort. Only 4 subjects (20%) in the RICE group and 3 subjects (15%) in the sham-RICE group experienced clinical deterioration, while 8 subjects (40%) in the RICE group and 5 subjects (25%) in the sham-RICE group experienced intracranial hemorrhage/oozing, but none of them were symptomatic. No other complications such as fall, angina, myocardial infarction, deep venous thrombosis, etc. occurred in either group. There were no significant differences between the groups in regard to the other safety endpoints except skin petechiae. Details of the primary outcomes are displayed in Table 2.

**Table 2:** Primary outcomes (safety) of the patients.

	**RICE (RIC** ** with exercise)** ** (*n =* 20)**	**Sham-RICE** ** (Sham-RIC** ** with exercise)** ** (*n =* 20)**	***P***
**Serious adverse events**			
RIC/RIC-sham related	0	0	
Exercise-related	0	0	
**Complications**			
Clinical deterioration	4 (20%)	3 (15%)	1.000
Intracranial hemorrhage/oozing	8 (40%)	5 (25%)	0.311
Recurrence of stroke	0	0	
Fall	0	0	
Angina	0	0	
Myocardial infarction	0	0	
Deep venous thrombosis	0	0	
Pulmonary embolism	0	0	
Pressure sore	0	0	
Chest infection	0	0	
Urinary tract infection	0	0	

### Efficacy

**Figure 2:**
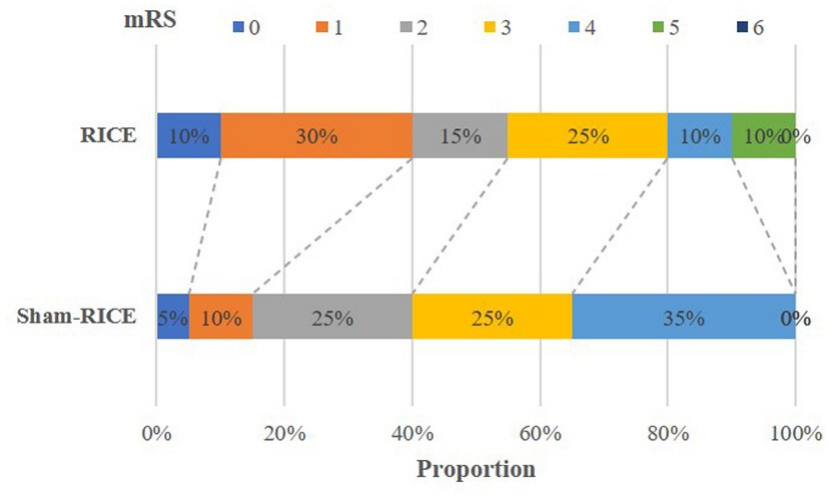
mRS shift: the percentage of patients achieving each mRS score at 90d.

The main efficacy outcome of this study was the percentage of patients at 90 days with a favorable outcome, which was defined as a score of 0 or 2 on the mRS (minimum or no disability). Although the difference in favorable outcome did not reach a significant level between the RICE group and the RICE-sham group after 90 days, the percentage of subjects with a favorable outcome was higher in the RICE group (55%) than in the sham-RICE group (40%) (Table 3). Also, the mRS shift data (percentage of patients achieving each mRS score after 90 days) revealed no difference in mRS between groups (Table 3 and Figure 2), and no significant differences were observed between the two groups in terms of NIHSS score, Barthel Index, and the proportion of patients achieving independent walking after 90 days (Holden functional classification of walking) (Table 3).

**Table 3:** Secondary outcomes (efficacy) of the patients at 90 days.

	**RICE (RIC** ** with exercise)** ** (*n =* 20)**	**Sham-RICE** ** (Sham-RIC** ** with exercise)** ** (*n =* 20)**	***P***
**Favorable outcome**			
mRS 0–2	11 (55%)	8 (40%)	0.342
**mRS category**			
0	2 (10%)	1 (5%)	
1	6 (30%)	2 (10%)	
2	3 (15%)	5 (25%)	
3	5 (25%)	5 (25%)	
4	2 (10%)	7 (35%)	
5	2 (10%)	0	
6	0	0	
**Other items**			
NIHSS score	4.35 ± 5.69	3.85 ± 3.12	0.732
Barthel index	71.5 ± 33.6	65.8 ± 32.6	0.586
**Holden functional classification of walking**			0.219
0	2	4	
1	1	2	
2	2	1	
3	1	1	
4	4	6	
5	10	6	

## Discussion

### Summary

This phase I study was designed to obtain safety and feasibility data for early RICE in patients with AIS. We found that RICE was well-tolerated and perfectly safe in the early stage of stroke, and there were no serious RICE related adverse events. All subjects in the RICE group completed the entirety of the experiment and follow-up. As described in our protocol (26), patients who received thrombolysis and/or endovascular treatments were enrolled in our study to accurately represent the standard of care for the patient population this rehabilitative strategy was designed for. We have considered these in our analyses to ensure that they did not confound our results.

### Possible side effects

The incidence of skin petechiae in the RICE group increased significantly (80% in RICE, 10% in sham-RICE), but all of participants completed their treatments without paleness of the skin, edema, or pain or tenderness in the distal region of the upper extremity. In the RICE arm, there seemed to be a higher rate of asymptomatic intracranial hemorrhage/oozing (40% in RICE vs. 25% in sham-RICE), but the difference did not reach a significant level. In this study, all of the patients with asymptomatic intracranial hemorrhage received intravenous thrombolysis, endovascular treatments, or bridging therapy. Furthermore, the hemorrhagic transformations were found by CT or MRI at 24 h after reperfusion therapies, suggesting that they were secondary to reperfusion therapies rather than the rehabilitative procedures. Although further studies with a larger sample size are needed to confirm this conclusion, the current study indicates that RICE was safe for AIS patients.

### Underlying mechanisms

RIC and exercise have several mechanisms that overlap, which include increased expression of heat shock proteins, enhanced involvement of the nitric oxide (NO) pathway, modification of Adenosine Triphosphate (ATP)-sensitive potassium (KATP) channels' functionality, enhanced antioxidant capacity, induction of autophagy, involvement of the opioid system, and regulation of the immune and inflammatory systems (24). They confer neuroprotection (29) and regeneration by enhancing angiogenesis (30), cerebral perfusion, cerebral collateral formation, and cerebral ischemia tolerance (31, 32). Further neuroprotection is established by reducing nerve injury (33), promoting nerve remodeling (30), and restoring function of paralyzed limbs (34). Because of these similarities, RIC is the perfect candidate as an adjunct therapy to exercise in the first 24h after an ischemic event. Hence, we hypothesized that the novel therapy, RICE, was a promising rehabilitation strategy for AIS. We predicted that the RICE intervention will improve motor recovery through the abovementioned mechanisms at the early optimal window for neural recovery. Our research and original findings suggest that RICE may have additional benefits when compared to RIC or exercise monotherapy in stimulating neuroplasticity, synaptogenesis, and angiogenesis after ischemic stroke (4). Furthermore, from a clinical perspective, the data suggests that RICE was beneficial for long-term functional improvements in sensorimotor, learning, and memory domains (26). Clearly, RICE may be a valid method to combine the strengths of both exercise and remote ischemic therapy. The present study suggests that RICE treatment may improve prognosis at 90 days, but it needs to be confirmed by large-scale randomized controlled studies.

### Limitations

This study has several limitations. First, this is a single-center study with a relatively small sample size, and the trend of improvement in clinical outcome was not powered to determine the efficacy of RICE in patients with AIS. Additionally, the patients recruited for this study were not completely representative of the stroke population: we limited the inclusion criteria to patients with moderate AIS and moderate-to-high disability rate (NIHSS scores between 6 and 16) (26). Therefore, the results may not be generalizable to all patients with AIS. An additional randomized and controlled multicenter trial study with a larger sample size covering the entire stroke population is necessary to establish RICE as a rehabilitative strategy.

In conclusion, these results suggest that RICE is safe and feasible for patients with AIS, but the current data was not powered to determine the efficacy of RICE. In a future study, we will further determine the efficacy of RICE in stroke patients in a multiple center RCT trial with a large sample size. We will also further explore potential mechanisms underlying this novel rehabilitation strategy.

## Data availability statement

The original contributions presented in the study are included in the article/supplementary material, further inquiries can be directed to the corresponding author/s.

## Ethics statement

The studies involving human participants were reviewed and approved by Medical Ethical Committee of Beijing Luhe Hospital, Capital Medical University. The patients/participants provided their written informed consent to participate in this study.

## Author contributions

YT, ZH, HD, and ZC performed the study, analyzed data, and prepared the manuscript. XG, YD, HL, and WK designed the study and revised the manuscript. FL, JG, and JL evaluated the subjects. All authors contributed to the article and approved the submitted version.

## Funding

This work was partially supported by the National Natural Science Foundation of China (82002382, 81871838, and 8200090876), the Youth Plan of Beijing Luhe Hospital (LHYY2021-LC08), the Science Foundation of Capital Medical University (PYZ21170), and the Yunhe Talent Program of Beijing Tongzhou District and the Laboratory Development Funds of Beijing Luhe Hospital (2022).

## Conflict of interest

The authors declare that the research was conducted in the absence of any commercial or financial relationships that could be construed as a potential conflict of interest.

## Publisher's note

All claims expressed in this article are solely those of the authors and do not necessarily represent those of their affiliated organizations, or those of the publisher, the editors and the reviewers. Any product that may be evaluated in this article, or claim that may be made by its manufacturer, is not guaranteed or endorsed by the publisher.
